# A quantitative cross-sectional study assessing the surgical trainee perception of the operating room educational environment

**DOI:** 10.1186/s12909-022-03825-6

**Published:** 2022-11-08

**Authors:** Neal Rupani, Ashish Evans, Mohammad Iqbal

**Affiliations:** 1 Nuffield Orthopaedic Centre, University of Oxford, Windmill Road, Oxford, OX3 7LD UK; 2 Royal Shrewsbury Hospital, Shrewsbury, England, UK

**Keywords:** Education environment, Surgery, Learning, Program evaluation, Education, Professional

## Abstract

**Background:**

Limited hours and service provision are diminishing training opportunities, whilst increasing standards of surgical proficiency is being sought. It is imperative to maximise the value of each educational event. An objective measure of higher surgical trainee perception of the operating room environment in England has not been performed before and this can steer future change in optimising educational events in theatre. The Operating Room Educational Environment Measure (OREEM) evaluates each component of the learning environment to enable optimisation of these educational events. However, the OREEM has not yet been assessed for reliability in higher surgical trainees in England.

The aim of the current study was to explore areas of strength and weakness in the educational environment in the operating room as perceived by surgical trainees’ in one English region. The secondary aim was to assess the reliability of the OREEM.

**Methods:**

Using a quantitative approach, data was collected over one month from surgical trainees in England using the OREEM.

**Results:**

Fifty-four surgical trainees completed the questionnaire. The OREEM had good internal consistency (α = 0.906, variables = 40). The mean OREEM score was 79.16%. Areas for improvement included better learning opportunities (average subscale score = 72.9%) and conducting pre- and post-operative teaching (average score = 70.4%). Trainees were most satisfied with the level of supervision and workload (average subscale score = 82.87%). The learning environment favoured senior trainees (*p* = 0.017). There was a strong correlation between OREEM and the global satisfaction score (*p* < 0.001).

**Conclusions:**

The OREEM was shown to be a reliable measure of the educational environment. It can be used to identify areas of improvement and as an audit tool. The current perception of the education environment is satisfactory, however, areas of improvement include reducing service provision, empowering trainees to plan lists, improving teamwork and using tools to optimise the educational value of each operation. There is a favourable attitude regarding the use of improvement tools, especially for dissatisfied trainees.

**Supplementary Information:**

The online version contains supplementary material available at 10.1186/s12909-022-03825-6.

## Educational objectives

- Opportunities for surgical trainees to enhance surgical skill are increasingly diminishing
- Maximising the value of each educational event is key to gain proficiency from surgical training
- The OREEM reliably evaluates each component of the learning environment
- The OREEM can identify areas of improvement in surgical training
- Pre-operative goal directed objective setting and delivery of post-operative feedback need improvement.

## Introduction

A critical objective of surgical training is to produce a surgeon who can practice competently and autonomously in the operating room [1]. The operating theatre is a focal point for this process. Traditionally, the Halstedian Apprenticeship model of “learning by doing” – where case volume equates to expertise – underpinned the principles of learning surgical skills [2].

Challenges have surfaced in how a trainee is expected to achieve operative competency in the face of a progressively growing operative repertoire and a heightened expected competency, all whilst being hampered by an abundance of barriers that limit training opportunities. There has been an erosion of training time and opportunities since the introduction of the 2003 European Working Time Directive [3, 4]. Restricted hours have led to a reduction in time to prepare for operations and reduced opportunities to attend emergency cases [5]. The service:education ratio has risen with trainees now spending only 6–14% of their training time operating [6]. There has been an increase in the number of clinical fellowships leading to a division of training time between the trainee and clinical fellow, thus further reducing training opportunities [7]. Increasing patient-expectations, programmes like Getting It Right the First Time (GIRFT), publication of surgeon-specific outcomes, medico-legal factors, and the inherent reduction in theatre efficiency that results from trainees performing operations have led to consultants performing rather than teaching surgery, and so reduced learning opportunities [8–10]. These barriers limit the application of the experiential learning model. Thus, there must be a shift from learning by quantity to learning with quality, and the operating room itself needs to be seen and addressed as an educational environment.

A consensus definition for the educational environment is not apparent, but one description of it is the climate that affects all aspects of learning within an educational setting [11]. It encompasses the physical environment, the characteristics of the involved participants, the strategies involved in teaching and the culture that infuses the educational environment. The educational environment dictates how, why and what trainees learn [12]. Therefore, improving the educational environment and the learner’s perception of the environment leads to better learning and outcomes [13–15]. However, Silen describes the operating room as inconducive to learning, hostile and a deterrent to surgical training [16]. That is owing to the operating room being a unique and dynamic environment, with minimal control and order. The optimal educational environment, which is one that allows the short and long term learning needs of the surgical trainees to be fulfilled, is formed by a framework that promotes teaching pre-, intra- and post-operatively [17]. Thus, effective teaching may be enhanced when emphasis is placed on preparation, intra-operative instruction and post-operative reflection in a supportive educational environment [18].

There is acknowledgement of the importance of a positive educational environment in fostering improved learner performance [19]. The inherent value of developing an instrument to assess the educational environment is derived from deconstructing and defining the imperative components of the educational environment, and targeting those modifiable components for improvement [11]. This has led to numerous instruments in the medical field being created to assess the educational environment. A 40-statement tool specific to the operating room was developed and validated by Cassar in 2004, named the Operating Room Educational Environment Measure (OREEM) [20]. Other objective tools have not been described in the literature. The full questionnaire can be seen in Additional file 1. The components of the OREEM were divided into four sub-scales: teaching and training, learning opportunities, atmosphere and supervision, workload and support.

The OREEM and its subscales were initially validated using a small sample of 26 pre-specialist trainees and have been further validated through studies worldwide [21–25]. This was a derivative of the Anaesthetic Trainee Educational Environment Measure, which as the first of these measures [26], but was adapted to be surgical training specific. A literature review analysis table outlining the current use of this tool can be seen in Additional file 2. The uses today have largely been observational studies worldwide in different training programs, where educational environments can not be assumed to be identical. Only one study to date has used this tool in a longitudinal study, to assess the impact of a learning tool [27]. However, importantly, the OREEM has yet to be explored in England on higher surgical trainees, which internationally are equivalent to surgical residents greater than half way through their training program.

This study will primarily evaluate surgical trainees’ objective perspectives of the current operating room educational environment in one English region. A secondary aim is to demonstrate the reliability of this tool within England, which has not been demonstrated prior. The primary aim will allow training programs to identify modifiable factors that could enhance the learning environment. The author of this study is aware that some components of the educational environment, which largely related to the physical and interpersonal factors, are not within the control of a trainee or trainer, and thus may not be immediately modifiable or require senior managerial intervention to lead to change. However, the author hopes these factors should be highlighted to the relevant training programmes for awareness with a view to foster improvement.

Furthermore, the author hopes to identify modifiable factors that remains in the control of the trainee and trainer. An example of this is where tools based on learning theory can be developed or implemented within the operating room to facilitate learning. They are aimed at addressing part of or the whole of the pre-operative, intra-operative and post-operative educational model, which is based on the three stages of learning coined Briefing, Intra-operative and Debriefing (BID) [28]. A tertiary aim is therefore to assess whether any educational learning tool is currently being used by trainees, and their attitude for implementing one to enhance learning.

### Personal ontology and epistemology

The author’s initial personal ontology and epistemology perspective were in line with a positivist paradigm. This paradigm is based on the belief that genuine knowledge is gained from observation and experimentation [29]. When looking at an educational environment measure, this is formed from numerical data, and therefore is conducive to aligning with the positivist researcher. If scientific etiquette is maintained, the author’s own cultural belief would not affect the outcome, and neutrality would be maintained. However, this does lead us to a fallacy of this paradigm, that although the paradigm is rooted in objectivity, the measurement is imperfect as human perception is flawed [30]. That is to say a participants assessment of the same learning environment may be different, and confounded by inner beliefs.

## Methods

### Study design, participants and sampling

A cross-sectional study design was implemented. Data were collected anonymously using Online Surveys, which was registered through the University of Edinburgh. It has General Data Protection Regulation (GDPR) compliance and meets the Information Security Standards [31, 32]. The survey contained demographic information of the participant, the OREEM tool, an 11-point numerical scale to assess global satisfaction, questions on awareness and current use of any educational tools, a further 11-point numeric scale to assess the trainee’s perception of the educational tool, and consent to be contacted for further interview. A participant information sheet was also provided. The 40-statement tool was scored on 5 point Likert scoring from 1–5 to allow comparison to previous studies, which all have not accounted for systematic overestimation [33].

This study only included surgical trainees within a single region within South East England. Distribution of the survey was done anonymously via departmental administrators via email, to maintain participant anonymity. A link was sent to each participant, with a participant information sheet of the study and a reminder at week 2 and week 4. All surgical specialties were included. Exclusion criteria included locum doctors; those averaging less than 2 half-day sessions per week in theatre; and trainees without a trainer with whom they routinely learn from. No incentives were provided to participants.

### Data statistical analysis

Prior to analysis, negatively phrased statements in the OREEM had their value reversed. SPSS version 24 was used for data analysis. Participants were divided into groups of ‘less experienced surgical trainees’ (ST5 and below) and ‘more experienced trainees’ (ST6 and above). The hospitals were divided into teaching hospitals and district general hospitals.

For the individual items of the OREEM statements, data were treated as ordinal data. The median of each score was used, as this is the preferred method for measuring central tendency within a Likert scale analysis [34]. Comparison between surgical trainee groups and OREEM scores and global satisfaction was analysed with the Mann Whitney U non-parametric test. The author acknowledges the debate as to whether summed Likert scales can be treated as parametric [35, 36]. However, in not knowing if the final sample size which affects the adequacy of parametric analysis, and that the other OREEM studies used non-parametric data, the author opted for this approach. Summated scores from the Likert scales were treated as interval data as per the central limit theorem, as increasing data points increases the tendency to normal distribution [37]. This is based on each item is measured on the same scale, and a preferred minimum of 8 items were used in the sum.

Cronbach’s α coefficient was used to assess the internal consistency of the OREEM and each subscale. An approach to consider using an “α if item deleted” strategy that would improve reliability was considered and would only be done in a limited manner if required.

A pre-hoc sample size analysis was performed. With a predicted sample size of 50 and 95% significance levels required, the confidence intervals would be set at a total of 8% of the total OREEM score.

### Ethics

The Health Research Authority tool for United Kingdom ruled out the requirement for Research Ethical Committee approval or the requirement for an Independent Research Application, in accordance with the national regulations outlined in the Standard Operating Procedures for Research Ethics Committees [38–40].

No stakeholders were gained that needed responsibilities assessed. No University sponsor was required after consultation with Academic and Clinical Central Office for Research and Development, University of Edinburgh. The study design met all the requirements of the Ethical Guidelines for Educational Research [41], and the following principles were maintained throughout.

#### Consent

Participants were given sufficient information on the nature of the project, the rationale for the project and what is involved in being a participant to allow them to make voluntary informed consent.

#### Transparency

Participants were made aware of the use of the data for further research projects, and that the research was being undertaken for an educational award.

#### Right to withdraw

All participants were made aware of their right to withdraw at any time up to the commencement of the analysis. No reason was required for withdrawal, and a receipt number was given to facilitate this if required.

#### Incentives

No incentives were provided to participants. Furthermore, the study is self-funded and based on the authors own motives with no organisational influence.

The risks of the study were limited due to minimisation methods such as anonimisation of the trainee and trainer.

#### Privacy and data storage

The research project was conducted in compliance with GDPR [31]. Data collected and downloaded for analysis was stored on a password protected NHS secure network and with data encryption if transferred. Emails were sent out by the each department’s administrator using the official NHS trust email servers.

## Results

### Demographics

One hundred forty trainees were contacted, of which 54 responded (38.6%), of which their data can be seen in Table 1. There was a male predominance in the sample (81.5%), in keeping with recent consensus data by the Royal College of Surgeons [42, 43].

**Table 1:** Demographics

Demographics	N (%)
Gender	
Male	44 (81.5)
Female	10 (19.5)
Years of Experience	
1	3 (5.6)
2	4 (7.4)
3	7 (13.0)
4	7 (13.0)
5	9 (16.7)
6	6 (11.1)
7	12 (22.2)
8	6 (11.1)
Hospital	
Tertiary Hospital	22 (40.7)
District General Hospital	32 (59.3)
Specialty	
Core Surgery	6 (11.1)
General Surgery	12 (22.2)
Otolaryngology	2 (3.7)
Plastic Surgery	5 (9.3)
Trauma & Orthopaedics	24 (44.4)
Urology	3 (5.6)
Vascular Surgery	2 (3.7)

The surgical training programme is 8 years. The mean length of time training was 4.72 years (SD = 2.05), split into junior (55.6%) and senior trainees (44.4%). Most trainees were in district general hospitals (59.3%). The authors could not find the national specialty distribution, but there is a skew towards Trauma and Orthopaedics (44.4%). Four sub-specialties had less than 10% representation.

### Internal consistency of summated OREEM score and OREEM subscales

Cronbach’s alpha analysis was performed to assess for internal consistency of the OREEM construct by two methods. The first was based on all 40 components (α = 0.906, variables = 40). The second was based on the four summated subscales (α = 0.810, variables = 4). Each subscale was assessed for reliability and transformed to comparable scales to ensure equal weighting prior to calculation. ‘If item deleted’ analysis did not drop the alpha score below 0.700. This demonstrates reliability of the OREEM construct.

Awareness is required that a very high alpha values, for α > 0.900, does not automatically imply a more reliable tool as the alpha coefficient tends to increase with the size of an instrument. This occurs due to the fact an item only needs to correlate with one other item to allow the overall value of alpha to be high and show internal consistency [44].

Cronbach’s alpha analysis was performed to demonstrate internal consistency of the four subscales; ‘Trainers and Teaching’ (α = 0.892, variables = 13), ‘Learning Opportunities’ (α = 0.737, variables = 11), ‘Atmosphere’ (α = 0.695, variables = 8) and “Supervisors, Workload and Support’ (α = 0.699, variables = 8). Post-hoc analysis was performed on the latter two subscales as they were within the debated acceptable criteria between α = 0.600–0.700 [45, 46]. ‘If item deleted’ analysis showed that α > 0.700 was achieved if items 26 or 27 from the ‘Atmosphere’ subscale or item 39 from the ‘Supervisors, Workload and Support’ subscale were removed. This supports the findings of reliability within the subscales.

The variance of the subscales within the whole measure demonstrated a 65.12% variance from a principal component, inferring unidimensionality of the OREEM construct. This means all the components were measuring the same homogenous single entity—the learning environment. The subject to variable ratio of 1.35 was not sufficient to allow further in-depth factor analysis [47].

### Overall satisfaction scores

Mean overall score was 158.3 (range = 118–190, SD = 16.88) out of 200, or 79.16%.

**Fig. 1:**
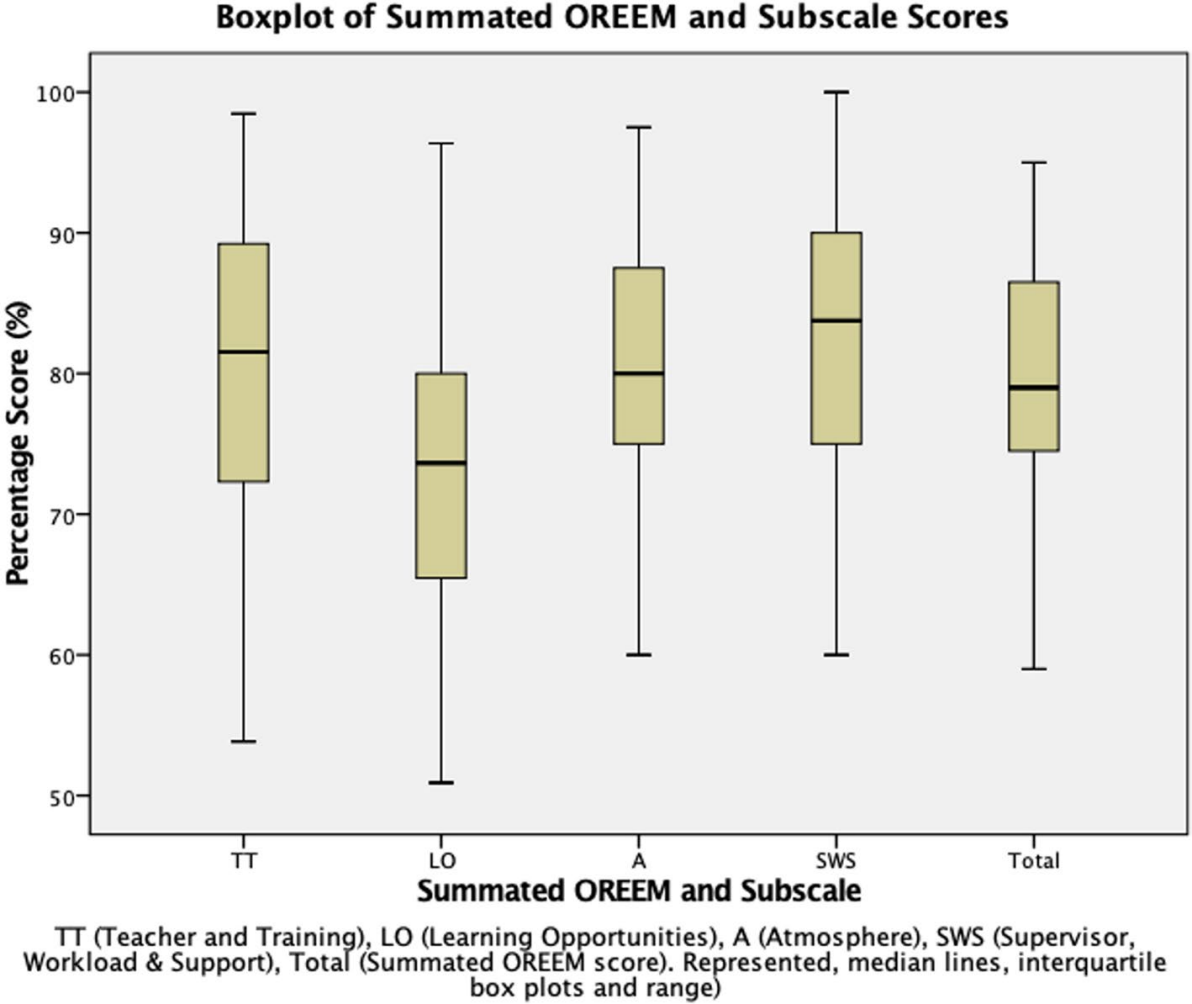
Boxplot of Summated OREEM and Subscale Scores

Figure 1 provides a summary of the summated OREEM and subscale median scores. This suggests similarity between subscales scores apart from ‘Learning Opportunties’, which scored lower at 72.90% (range = 50.91–96.36, SD = 11.09). The highest subscale score was ‘Supervisors, Workload and Support’ at 82.87% (range = 60–100, SD = 10.01), followed by ‘Atmosphere’ at 81.39% (range = 60–97.5, SD = 8.94), ‘Teachers and Training’ at 80.80% (range = 53.85–98.46, SD = 11.35).

**Fig. 2:**
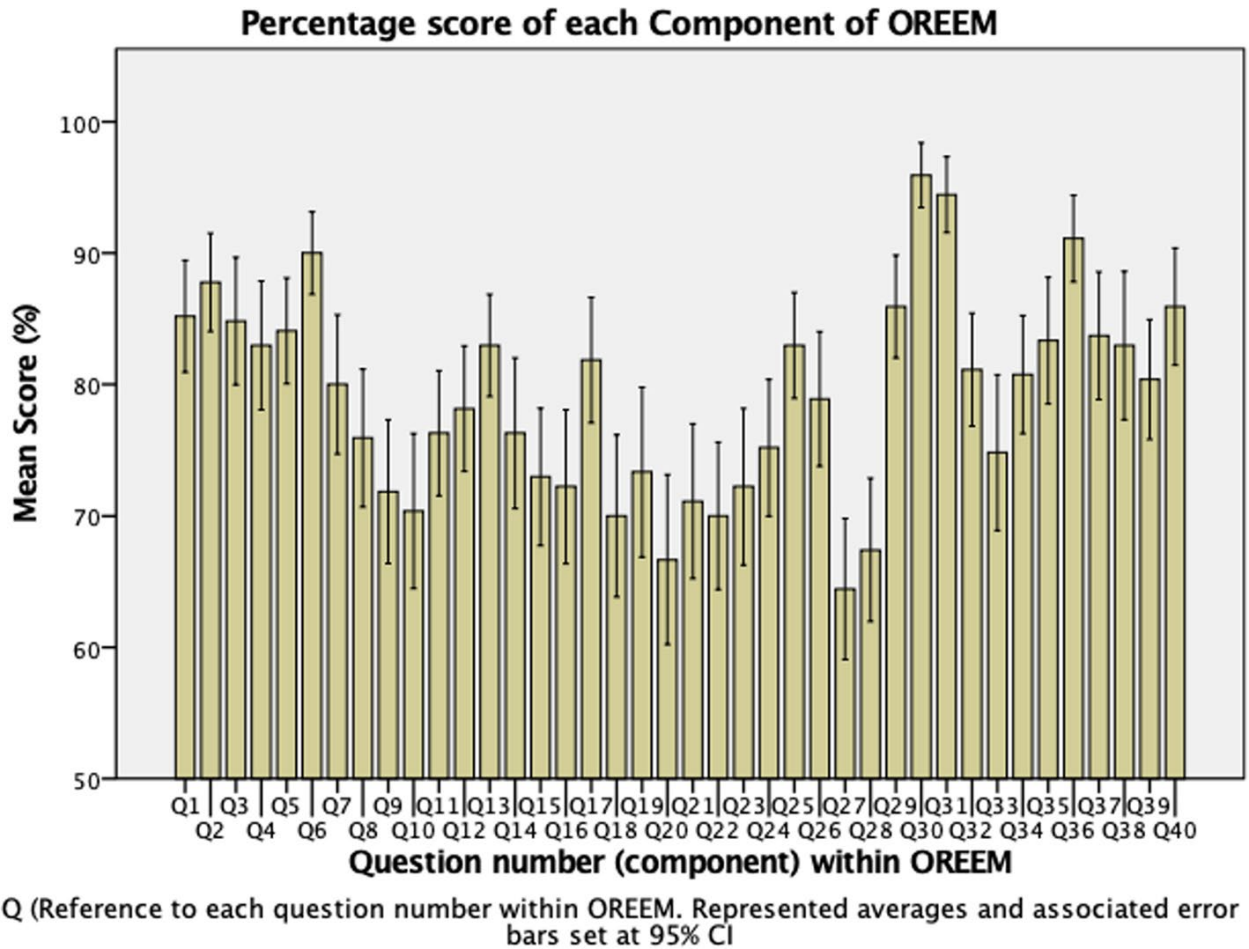
Percentage Score of each OREEM Component

Figure 2 shows the mean score as a percentage of each item with confidence intervals. Three items had a mean score < 70%—items 20 (66.67%), 27 (64.40%) and 28 (67.40%). These reflect hostility from other staff in the learning environment and lack of exposure to emergency cases. Two of these items are within the ‘Learning Opportunities’ subscale, which can help explain the boxplot in Fig. 1. Three items had a mean score > 90%—items 30 (96.00%), 31 (94.40%) and 36 (91.20%). These reflect a lack of gender or racial discrimination, as well an absence of feeling unsupported or out of a trainee’s depth.

### Global Satisfaction Score (GSS)

**Fig. 3:**
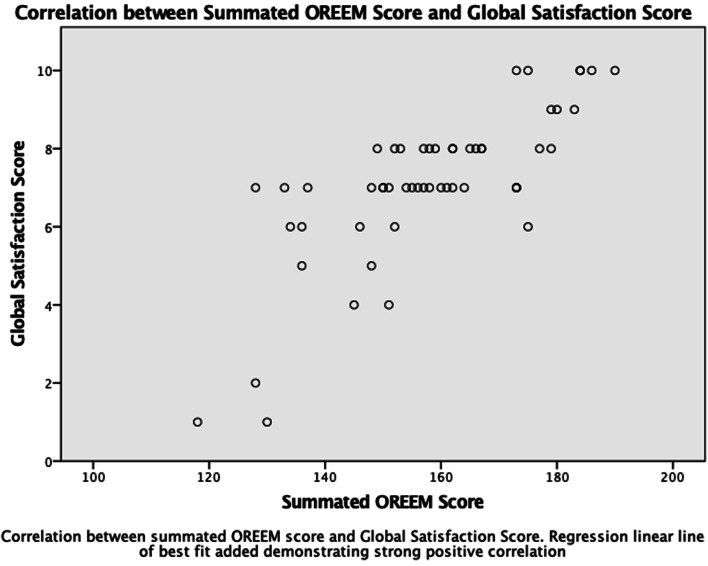
Correlation between Summated OREEM Score and GSS

Mean GSS was 8.11 (SD = 1.978). Figure 3 shows that as the summated OREEM score increased, so did the GSS. A strong positive correlation coefficient between the GSS and summated OREEM score was determined using Spearman’s rho (*r* = 0.755, *p* < 0.001). Correlation between the four subscales and the GSS showed moderate positive correlation between three of the subscales: ‘Trainers and Teaching’ (*r* = 0.668, *p* < 0,001); ‘Atmosphere’ (*r* = 0.571, *p* < 0.001) and ‘Learning Opportunities’ (*r* = 0.603, *p* < 0.001).

### Subgroup analysis

The Mann Whitney U test was utilised for subgroup analysis, and scores can be seen in Additional file 3. There was no significant difference between gender when analysing the total OREEM summated score, subscale scores, or GSS. Items 8 (U = 123, *p* = 0.027) and 9 (U = 132, *p* = 0.041) demonstrated the only significant difference with females scoring higher in both items relating to improved pre- and intra-operative training behaviours.

There was no significant difference between tertiary and district general hospitals when analysing the total OREEM summated or subscale scores, or GSS. However, items 25 (U = 232.5, *p* = 0.017), 33 (U = 235.0, *p* = 0.029), and 38 (U = 205.5, *p* = 0.002) demonstrated a difference with tertiary hospital trainees scoring higher. This was due to trainees’ perceived lack of opportunities due to service requirements. However, this was not reflected in a significant difference in the ‘Learning Opportunities’ subscale.

There was a statistically significant difference between junior and senior trainees with the total OREEM score (U = 223.5, *p* = 0.017), GSS (U = 188.5, *p* = 0.002) and ‘Learning Opportunities’ (U = 210.0, *p* = 0.008), ‘Atmosphere’ (U = 243.5, *p* = 0.041), and ‘Supervisors, Workload and Support’ subscales (U = 211.0, *p* = 0.009). Ten questions showed significant differences in favour of the senior trainees.

### Improvement tools

17/54 trainees were aware of an educational improvement tool that allows them to maximise each of their educational opportunities. 11 of these trainees actually use one in their current practice. Assessing the trainee perception of the benefits of such a tool scored a mean value of 6.83 (SD = 1.95) on an 11-point Likert scale.

There was a statistically significant moderate negative correlation, using Spearman’s Rho, between the OREEM summated score and assessment of the value of an improvement tool (*r* = -0.430, *p* = 0.001). This suggests trainees who perceive the educational environment negatively would more likely adopt a tool to improve their learning and educational opportunties. No other moderate to strong correlation was seen.

## Discussion

The quality of the learning environment is crucial to education, and attainment is positively correlated to the trainee’s perception of it [19]. It is for this reason that the Royal College of Surgeons have emphasised the importance of its improvement [15, 48].

The summated OREEM score from this study was 158.3/200, or 79.16%. Compared to seven of the OREEM studies, this group of trainees had a higher level of satisfaction with their learning room environment than international programmes.

The score that defines a good learning environment for trainees is yet to be defined [20, 49]. Cassar felt that a score over 75% demonstrated a satisfactory learning environment, however, the rationale for this threshold was not given. Kanshiro suggested a positive learning environment be defined with a score above 80%, as anything worse would be less than ‘agreeable’ on the OREEM Likert scale descriptors. This would suggest the learning environment for surgical trainees within this region is on the borderline for being acceptable to trainees.

A further consideration is the inflated value, when using a Likert score coded from 1–5 as opposed to 0–4, which has been what is used for all studies with OREEM to date. This is owing to the minimum score possible being 20%, and how low scores may be artificially inflated in what is termed ‘systematic overestimation’. [33] A score of 75% would be an over-estimation by 6.4% in prior studies.

The highest subscale score was from ‘Supervisors, Workload and Support’ at 82.87%, which has also been shown in half the OREEM studies internationally [24, 25, 50]. The lowest subscale score was ‘Learning Opportunities’, which was also found to be a substantial source of relative dissatisfaction within this and other studies [20, 22, 25]. Furthermore, this subscale had many items showing suboptimal satisfaction, related to not having enough opportunity to attend theatre (items 18, 19, 23), not having the right case mix (items 15, 16), and an inability to have sufficient emergency cases exposure (items 20, 21, 22).

The lack of concern regarding suitable workloads but with unsatisfactory ‘Learning Opportunities’, implies that trainees do not feel over-worked, rather that their training:service ratio is unfavourably skewed. This would add credence to the Temple Report to minimise service requirements to allow sufficient training time within normal working hours [51]. Causes for the lack of opportunity are likely the numerous barriers outlined in this article’s introduction. Clearly a balance will always need to be met between training and service provision. However, these authors believe that this source of dissatisfaction can only be positively addressed with institutional or national change. This could be in the form of protected theatre time, improved staffing numbers, and empowering trainees to participate in theatre list management.

Regarding emergency case exposure, this was suboptimal in all studies. Emergency cases are specifically highlighted to have an associated poor learning environment [16]. This is owing to stress in the trainer, a lack of exposure and numerous other external factors. The solution to this is not evident, and the degree of learning during these cases will largely be case dependent. Additional learning methods, such as simulation, have already started to be incorporated into curricula to tackle these specific cases where ‘on-the-job’ learning is not feasible [52].

In the ‘Teaching and Training’ component, given the importance of teaching procedural skills in a planned fashion, the use of pre-operative learning and objective setting (items 9, 10), was less than satisfactory. Aligning objectives and improving pre-operative procedural rehearsal in improving outcomes is seldom done but is shown to improve achievement of learning outcomes [53, 54]. Only 18% of trainees in America report that operative goals are identified, and 35% discuss operative plans prior to surgery [55]. As such, improvement in this area offers a low cost and valuable route to improved training.

Only two items from the ‘Atmosphere’ subscale demonstrated less than agreeable satisfaction, and were focused on hostility from staff due to the increased time taken for an operation (items 27, 28). The authors believe that awareness and education of all staff members in the training requirements and responsibilities of future surgeons would help to mitigate this negative learning behaviour. However, changing culture is difficult and time consuming and improvements from this are likely to be long-term goals. Furthermore, this hostility was also seen in half of the OREEM studies, suggestive of how widespread and difficult it would be to consistently and reproducibly change culture on a national scale [20, 22, 24].

### Subgroup analysis

The absence of contrasting experiences between male and female trainees differs from existing literature, as recent studies demonstrate half of female surgeons reporting gender discrimination in the operating room and being offered less autonomy [56, 57]. These studies were not conducted in England, so the lack of difference may be explained by the recent implementation and benefits of initiatives and heightened awareness in tackling this discrimination from organisations such as the Royal College of Surgeons [58]. These initiatives include increasing career flexibility, female role models and Women in Surgery programmes.

Comparison by type of hospital yields varying results in the existing literature and is further complicated by variability at institutional and international level, based on the set up and role of surgical trainees in each hospital, region and country. Future differences identified in this comparison may be valuable in promoting a culture where learning between hospitals can occur in how to improve the learning environment.

There was a significant difference favouring more experienced trainees within the summated OREEM score, as well as three of the four subscales. The only subscale which showed equipoise was “Trainers and Teaching” suggesting that trainer’s interaction remained equivalent irrespective of the trainee’s grade. However, the learning environment is otherwise not optimised for junior trainees. This is likely due to junior trainees having less opportunities due to managing other workloads, a more hostile environment due to operations taking longer and lower expectations meaning fewer opportunities [55]. Snyder also found that satisfaction directly correlated with length of time in training, and that there was a greater perception of performing a procedure in more senior trainees. This is due to graduated autonomy and less trainer ‘interference’ that occurs with increasing levels of experience.

### Reliability anaylsis

The OREEM demonstrated a good internal consistency as a single unidimensional measure of the learning environment [45, 46]. This is consistent with the other studies with a range of α = 0.793–0.97. Thus, OREEM can be utilised to assess trainees’ perceptions of the operating room learning environment in England. Caution to this finding however, must be made as with high-item number surveys, this can artificially raise the alpha value above 0.900.

All OREEM subscales demonstrated internal consistency, with a range of α = 0.695–0.892. Two of the four subscales were borderline on Streiner’s threshold for acceptable consistency, which is set at α = 0.700. However, they are comparable to other accepted learning environment measures not specific to the operating room [46, 59, 60]. This suggests that each subscale reliably demonstrates its underlying subscale construct, and that each subscale is measuring a different facet of the learning environment owing to its differing alpha coefficients. In all OREEM studies, the alpha coefficient for ‘Trainers and Teaching’ was highest, however, redundancy has previously been excluded within this subscale by exploratory factor analysis [22].

### Limitations

This was a single time-frame study leading to potential time-period or seasonal bias, depending on whether a surgical trainee was near the beginning or end of their rotation. This is likely to influence perceptions as more time with a trainer affords more opportunities as the trainee develops more proficiency. A further limitation was the study was limited to one region, meaning that the attitude to training may be different in other regions within the country and influenced by regional factors such as availability of trainees to Major Trauma Centres. 2019-nCov led to the dramatic reduction of operations and so learning opportunities. This significantly reduced learning opportunities at the time, and its effects on the operating environment post resumption is currently unclear. An element of respondent bias is likely to have impacted this study also. The study met the criteria to be suitably powered to show the desired effect. The response rate was 38.6%, which despite being a lower response rate when compared to the other OREEM studies, this is actually higher than what is expected from an online survey. This leads the author to believe that the other studies were not done in an online manner and subjects those studies to respondent bias.

## Conclusion

The operating room educational environment is currently satisfactory, and equivalent to other international training programmes. The level of current surgical trainee supervision and support is good. However, the areas of the learning environment that needs to be improved upon were both internal and external factors of the operating room learning environment. External factors related to a lack of opportunity due to workload, service provision and not enough theatre time, and need to be addressed at a local and regional level. Internal factors were related to a lack of pre-operative goal directed learning and post-operative feedback, and two areas where surgical trainees believe tools that guide learning would be beneficial to learning.

This reliability of this tools allows this study to serve as a pilot study for a national study to occur. This will help identify any areas or hospitals of excellence, where optimal surgical training and learning environments can be modelled on.

These authors believe the tool could also form an important quality assessment of the learning environment by individual hospitals or regional training programmes. This would allow identification and prioritisation of potentially modifiable factors that would enhance surgical training. Serial auditing with the OREEM following implementation of change, can assess the success of such change as well as ensuring high standards are being maintained over time.

Further longitudinal studies using OREEM should be conducted to identify if learning tools are implemented in to each educational event, whether this improves the educational environment. Further studies could also include a national mixed methods exploratory sequential typology study using OREEM to identify hospitals with the most conducive environments and elicit the major differential factors that lead to this.

## Supplementary Information

**Additional file 1.** OREEM Questionnaire.

**Additional file 2.** Literature Review Analysis Table.

**Additional file 3: Table 1.** Subgroup Non-parametric Analysis for Differences.

## Data Availability

The datasets used and/or analysed during the current study are available from Neal Rupani, the corresponding author, on reasonable request, but restrictions apply to the availability of these data, which were used under license for the current study, and so are not publicly available.
