# Supplementary figures and images for: A quantitative cross-sectional study assessing the surgical trainee perception of the operating room educational environment
Source: BMC Med Educ. 2022 Nov 8;22:764. doi: 10.1186/s12909-022-03825-6 (PMC9640905; doi:10.1186/s12909-022-03825-6)

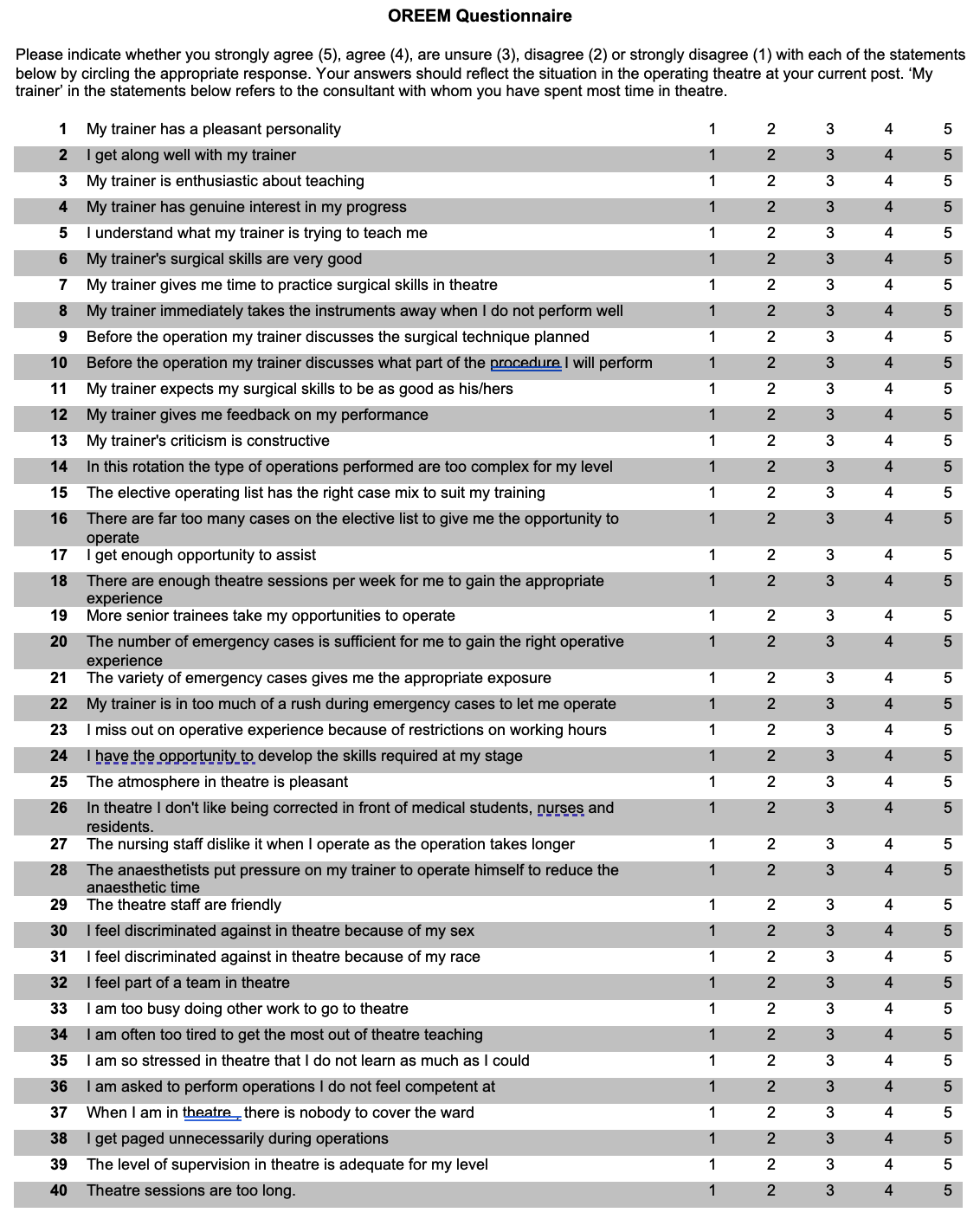


*Additional file 1: OREEM Questionnaire*

Supplement: Supplementary file 1 — Additional file 1. OREEM Questionnaire. [file 12909_2022_3825_MOESM1_ESM.docx]
