# Supplementary material for: A quantitative cross-sectional study assessing the surgical trainee perception of the operating room educational environment
Source: BMC Med Educ. 2022 Nov 8;22:764. doi: 10.1186/s12909-022-03825-6 (PMC9640905; doi:10.1186/s12909-022-03825-6)
Supplement: Supplementary file 2 — Additional file 2. Literature Review Analysis Table. [file 12909_2022_3825_MOESM2_ESM.docx]

## *Additional file* 2 – Literature Review Analysis Table

| **Author/s & Year** | **Aim(s)/**  **Questions** | **Design** | **Population/ Sample** | **Methods** | **Analysis** | **Major**  **Findings** | **Limitations/issues** |
| --- | --- | --- | --- | --- | --- | --- | --- |
| Cassar (2004) | To develop and validate an Educational Environment Measure for the Operating Room | Mixed methods | Consultants (n=7) for qualitative review  Basic surgical residents at Aberdeen Basic Surgery Rotation (n=26) | Literature review and exploratory interviews n=7)  Questionnaire (n=26) | Reliability analysis (Cronbach alpha co-efficient) & ANOVA | A valid and reliable Educational Measure  Main tool  Restrictive factors is learning opportunities | Limited participants as pilot study.  Single centred study  Failure to identify hidden educational events from trainees |
| Kanashiro (2006) | To validate the OREEM tool for use in North America  To evaluate and pilot the tool, and assess the environment at the local institution, and demographic bias. | Cross-sectional survey | General surgery residents at one institution between November 2013 to January 2014 in Calgary, Canada | Questionnaire (n=22/23) | Mann-Whitney U  Cronbach ahpla co-efficient for reliability analysis | 74% OREEM score labelled as satisfactory  A valid and reliable educational measure  Decreased satisfaction in female population.  Poor pre-operative briefing. | Limited participants as pilot study.  Single centred study  Failure to identify qualitative data |
| Mahoney (2010) | To validate the STEEM tool for Australasia  To assess trainee satisfaction of the educational operating room environment | Mixed:  Cross-sectional survey  Qualitative | All residents registered to Royal Australisian College of Surgery | Questionnaire (n=365/1500) | Mann-Whitney U  Spearman’s Rho  Cronbach ahpla co-efficient for reliability analysis | A valid and reliable research tool  Qualitative assessment highlighted areas to improve. | Poor return rate  Demographic data not collected, potential to bias |
| Diwadkar (2010) | To identify differences in perception between junior and senior trainees  To validate the tool for internal consistency and reliability | Cross-sectional survey | Obstetric and Gynaecology trainees in Ohio across 3 centres | Questionnaire (n=24/25) | Cronbach ahpla co-efficient for reliability analysis  ANOVA  Shapiro-Wilks | A valid and reliable research tool  Different perceived environments across different sites | Small sample size  Single time frame – variability may exist at different points of the academic year |
| Snyder (2012) | To evaluate surgeons perceptions of teaching in the operating room via specific educational interactions.  To identify opportunities of improving operating room teaching. | Cross-sectional survey | All residents under Accreditation Council of General Medical Education in America  n = 4926 from May to September 2010 | Questionnaire (n=998/4926) | Pearson X^2^ Test | 84% offer intra-operative advice.  Only 18% undertake pre-operative briefing Only 37% undergo post-operative debrief on positive feedback and future objectives  Resident’s perception they are not performing cases despite logging them as so. | Despite large sample site, poor return rate of questionnaire (non-responder bias).  Non-validated questionnaire.  No faculty perception, with plausible hidden interactions not identified as teaching by trainees |
| Al Sheikh (2012) | To assess perception of learning environment in Saudi Arabia, any gender discrepancy and for correlation with perception and academic success | Cross-sectional survey | Medical students in Saudi Arabia single University | Questionnaire (n=91/145) | Cronbach alpha co-efficicient for reliability analysis  Spearman’s rho | Valid and reliable tool  Gender difference in perception biased unfavourably against female gender  No correlation between academic success | Limited to medical students – different population  Single centred |
| Binsaleh (2015) | To evaluate the theatre learning environment in Saudi Arabia.  To assess learning environment perception compared to stage of training. | Cross-sectional survey | Urology residents in Saudi Arabia | Questionnaire (n=33/72) | Mann-Whitney U/Kruskal-Wallis  Cronbach ahpla co-efficient for reliability analysis | 68% STEEM score, which has been determined ‘less than ideal’. Comparative  No statistical significant difference in perception at different stages of training | Small sample size  Gender bias owing to lack of female in training  Low (45% response rate)  Comparative results to other countries, but assessed as ‘less than ideal’ |
| Fryer (2016) | To assess whether trainee satisfaction and operating time changed with implementation of a autonomy and supervision assessment system | Cross sectional survey  Quasi-experimental | Consultants and resident at single centre in Chicago, America | Questionnaire (58) | Wilcoxin | No change in OREEM or operative time using systems to log autonomy and supervision ratings | Single centred  Low clinical value as expensive IT infra-structure required  Unable to match pre- and post- surveys |
| Al Ramsi (2019) | Explore perceptions of O&G residents, and compare between two different hospitals | Cross-sectional survey | O&G Residents in UAE across 2 centres | Questionnaire (n=31/34) | Cronbach alpha co-efficicient for reliability analysis  t-test | A valid and reliable tool  Comparative positive correlation to other studies  Correlation of each subscale | Small sample  No qualitative research |
