# Supplementary material for: A quantitative cross-sectional study assessing the surgical trainee perception of the operating room educational environment
Source: BMC Med Educ. 2022 Nov 8;22:764. doi: 10.1186/s12909-022-03825-6 (PMC9640905; doi:10.1186/s12909-022-03825-6)
Supplement: Supplementary file 3 — Additional file 3: Table 1. Subgroup Non-parametric Analysis for Differences. [file 12909_2022_3825_MOESM3_ESM.docx]

Table 1: Subgroup Non-parametric Analysis for Differences

|  | Gender | | | | Hospital Type | | | | Junior vs Senior Trainees | | | |
| --- | --- | --- | --- | --- | --- | --- | --- | --- | --- | --- | --- | --- |
|  | U score | Sig. (2-tailed) | Male | Female | U score | Sig. (2-tailed) | Tertiary | DGH | U score | Sig. (2-tailed) | Junior | Higher |
| OREEM | 191.5 | 0.535 | 157.5 | 164.5 | 258.0 | 0.099 | 166.5 | 157.0 | 223.5 | 0.017 | 155.5 | 165.5 |
| TT | 212.5 | 0.874 | 52.5 | 53.0 | 303.0 | 0.393 | 53.5 | 51.5 | 287.5 | 0.210 | 51.0 | 53.5 |
| LO | 161.0 | 0.193 | 39.0 | 42.5 | 276.5 | 0.186 | 42.0 | 39.0 | 210.0 | 0.008 | 38.5 | 42.5 |
| A | 186.5 | 0.462 | 32.0 | 33.5 | 271.5 | 0.156 | 34.0 | 32.0 | 243.5 | 0.041 | 32.0 | 34.0 |
| SWS | 170.0 | 0.270 | 33.0 | 35.5 | 242.5 | 0.053 | 34.0 | 33.0 | 211.0 | 0.009 | 32.0 | 35.5 |
| GSS | 192.0 | 0.530 | 8.0 | 8.5 | 319.0 | 0.555 | 8.0 | 8.0 | 188.5 | 0.002 | 8.0 | 9.0 |
| Q1 | 196.0 | 0.550 | 4.0 | 4.0 | 336.5 | 0.769 | 4.0 | 4.0 | 322.5 | 0.490 | 4.0 | 4.0 |
| Q2 | 210.0 | 0.882 | 4.5 | 4.0 | 321.0 | 0.557 | 5.0 | 4.0 | 357.5 | 1.000 | 4.5 | 4.0 |
| Q3 | 198.0 | 0.627 | 4.0 | 5.0 | 314.0 | 0.478 | 5.0 | 4.0 | 355.5 | 0.954 | 4.0 | 4.5 |
| Q4 | 207.0 | 0.796 | 4.0 | 4.0 | 325.0 | 0.645 | 4.0 | 4.0 | 338.0 | 0.698 | 4.0 | 4.0 |
| Q5 | 185.5 | 0.412 | 4.0 | 4.0 | 326.5 | 0.631 | 4.0 | 4.0 | 334.0 | 0.651 | 4.0 | 4.0 |
| Q6 | 202.5 | 0.758 | 5.0 | 4.5 | 297.5 | 0.293 | 5.0 | 4.0 | 306.5 | 0.303 | 4.0 | 5.0 |
| Q7 | 218.0 | 0.976 | 4.0 | 4.0 | 346.0 | 0.923 | 4.0 | 4.0 | 299.5 | 0.273 | 4.0 | 4.0 |
| Q8 | 123.0 | 0.027 | 4.0 | 4.5 | 318.0 | 0.559 | 4.0 | 4.0 | 299.0 | 0.265 | 4.0 | 4.0 |
| Q9 | 132.0 | 0.041 | 4.0 | 3.0 | 263.5 | 0.101 | 4.0 | 4.0 | 286.0 | 0.177 | 4.0 | 4.0 |
| Q10 | 157.5 | 0.159 | 4.0 | 3.0 | 278.0 | 0.173 | 4.0 | 4.0 | 231.5 | 0.018 | 3.0 | 4.0 |
| Q11 | 205.0 | 0.752 | 4.0 | 4.0 | 329.0 | 0.670 | 4.0 | 4.0 | 256.0 | 0.049 | 4.0 | 4.0 |
| Q12 | 218.5 | 0.965 | 4.0 | 4.0 | 314.0 | 0.496 | 4.0 | 4.0 | 301.5 | 0.288 | 4.0 | 4.0 |
| Q13 | 196.0 | 0.602 | 4.0 | 4.0 | 316.0 | 0.490 | 4.0 | 4.0 | 251.0 | 0.037 | 4.0 | 4.0 |
| Q14 | 175.5 | 0.316 | 4.0 | 4.5 | 342.5 | 0.874 | 4.0 | 4.0 | 302.0 | 0.299 | 4.0 | 4.0 |
| Q15 | 170.0 | 0.247 | 4.0 | 4.0 | 337.0 | 0.783 | 4.0 | 4.0 | 289.0 | 0.187 | 4.0 | 4.0 |
| Q16 | 193.0 | 0.562 | 4.0 | 3.0 | 343.0 | 0.880 | 4.0 | 4.0 | 352.5 | 0.905 | 4.0 | 4.0 |
| Q17 | 172.5 | 0.256 | 4.0 | 4.0 | 304.0 | 0.381 | 4.0 | 4.0 | 331.5 | 0.578 | 4.0 | 4.0 |
| Q18 | 220.0 | 1.000 | 4.0 | 4.0 | 280.0 | 0.195 | 4.0 | 3.5 | 322.0 | 0.501 | 4.0 | 4.0 |
| Q19 | 193.5 | 0.552 | 4.0 | 4.5 | 329.0 | 0.682 | 4.0 | 4.0 | 216.0 | 0.009 | 3.5 | 4.0 |
| Q20 | 147.5 | 0.100 | 3.0 | 4.0 | 302.5 | 0.371 | 4.0 | 3.0 | 273.0 | 0.121 | 3.0 | 4.0 |
| Q21 | 142.0 | 0.063 | 4.0 | 4.0 | 307.0 | 0.406 | 4.0 | 4.0 | 283.5 | 0.158 | 4.0 | 4.0 |
| Q22 | 160.0 | 0.165 | 4.0 | 4.0 | 282.5 | 0.202 | 4.0 | 4.0 | 207.5 | 0.005 | 3.0 | 4.0 |
| Q23 | 192.5 | 0.558 | 4.0 | 4.0 | 266.0 | 0.120 | 4.0 | 3.5 | 300.0 | 0.286 | 4.0 | 4.0 |
| Q24 | 162.0 | 0.176 | 4.0 | 4.0 | 264.5 | 0.092 | 4.0 | 4.0 | 269.0 | 0.082 | 4.0 | 4.0 |
| Q25 | 199.5 | 0.692 | 4.0 | 4.0 | 232.5 | 0.017 | 5.0 | 4.0 | 256.5 | 0.040 | 4.0 | 4.0 |
| Q26 | 189.5 | 0.481 | 4.0 | 4.0 | 283.5 | 0.203 | 4.0 | 4.0 | 345.5 | 0.798 | 4.0 | 4.0 |
| Q27 | 146.5 | 0.092 | 3.0 | 4.0 | 285.0 | 0.219 | 3.5 | 3.0 | 234.0 | 0.020 | 3.0 | 4.0 |
| Q28 | 214.5 | 0.920 | 4.0 | 4.0 | 335.5 | 0.759 | 4.0 | 4.0 | 289.0 | 0.188 | 3.5 | 4.0 |
| Q29 | 177.0 | 0.291 | 4.0 | 5.0 | 290.0 | 0.231 | 4.5 | 4.0 | 333.0 | 0.608 | 4.0 | 4.0 |
| Q30 | 185.5 | 0.236 | 5.0 | 5.0 | 295.0 | 0.152 | 5.0 | 5.0 | 319.5 | 0.370 | 5.0 | 5.0 |
| Q31 | 200.5 | 0.534 | 4.0 | 4.0 | 287.0 | 0.161 | 5.0 | 5.0 | 308.0 | 0.269 | 5.0 | 5.0 |
| Q32 | 218.5 | 1.000 | 4.0 | 4.0 | 260.5 | 0.087 | 4.0 | 4.0 | 252.0 | 0.038 | 4.0 | 4.0 |
| Q33 | 207.5 | 0.782 | 4.0 | 4.0 | 235.0 | 0.029 | 4.0 | 4.0 | 203.5 | 0.003 | 4.0 | 4.0 |
| Q34 | 157.0 | 0.148 | 4.0 | 5.0 | 331.0 | 0.693 | 4.0 | 4.0 | 323.5 | 0.503 | 4.0 | 4.0 |
| Q35 | 163.0 | 0.196 | 4.0 | 5.0 | 324.0 | 0.611 | 4.0 | 4.0 | 285.0 | 0.171 | 4.0 | 4.5 |
| Q36 | 166.0 | 0.220 | 5.0 | 5.0 | 320.5 | 0.547 | 5.0 | 5.0 | 333.0 | 0.588 | 5.0 | 5.0 |
| Q37 | 208.5 | 0.789 | 4.0 | 4.0 | 249.5 | 0.054 | 5.0 | 4.0 | 244.5 | 0.031 | 4.0 | 5.0 |
| Q38 | 201.0 | 0.664 | 4.0 | 5.0 | 205.5 | 0.005 | 5.0 | 4.0 | 224.0 | 0.010 | 4.0 | 5.0 |
| Q39 | 219.0 | 0.992 | 4.0 | 4.0 | 311.0 | 0.468 | 4.0 | 4.0 | 319.0 | 0.450 | 4.0 | 4.0 |
| Q40 | 172.0 | 0.267 | 4.0 | 5.0 | 323.0 | 0.591 | 4.5 | 4.0 | 326.0 | 0.527 | 4.0 | 4.5 |
